# Individual-Level Fatality Prediction of COVID-19 Patients Using AI Methods

**DOI:** 10.3389/fpubh.2020.587937

**Published:** 2020-09-30

**Authors:** Yun Li, Melanie Alfonzo Horowitz, Jiakang Liu, Aaron Chew, Hai Lan, Qian Liu, Dexuan Sha, Chaowei Yang

**Affiliations:** ^1^Department of Geography and Geoinformation Science, George Mason University, Fairfax, VA, United States; ^2^National Science Foundation (NSF) Spatiotemporal Innovation Center, George Mason University, Fairfax, VA, United States; ^3^Department of Biophysics, Johns Hopkins University, Baltimore, MD, United States; ^4^Marian and Rosemary Bourns College of Engineering, University of California, Riverside, Riverside, CA, United States; ^5^Valencia High School, Yorba Linda, CA, United States; ^6^Department of Geographical Sciences, University of Maryland, College Park, MD, United States

**Keywords:** COVID-19, machine learning, deep learning, pandemic, rare event, fatality prediction

## Abstract

The global covid-19 pandemic puts great pressure on medical resources worldwide and leads healthcare professionals to question which individuals are in imminent need of care. With appropriate data of each patient, hospitals can heuristically predict whether or not a patient requires immediate care. We adopted a deep learning model to predict fatality of individuals tested positive given the patient's underlying health conditions, age, sex, and other factors. As the allocation of resources toward a vulnerable patient could mean the difference between life and death, a fatality prediction model serves as a valuable tool to healthcare workers in prioritizing resources and hospital space. The models adopted were evaluated and refined using the metrics of accuracy, specificity, and sensitivity. After data preprocessing and training, our model is able to predict whether a covid-19 confirmed patient is likely to be dead or not, given their information and disposition. The metrics between the different models are compared. Results indicate that the deep learning model outperforms other machine learning models to solve this rare event prediction problem.

## Introduction

The Coronavirus (SARS-CoV-2 virus) has caused detrimental effects since its inception in late 2019. In the months since, the virus has progressed to become a widespread global pandemic. Over two hundred countries (tracked by Worldometer^1^) have been plagued by the virus, leading to almost a total of 530,000 deaths worldwide, as of July 5th, 2020 (1). Not only has this virus gravely affected individuals who have contracted the infection, but also healthcare employees and even patients with illnesses unrelated to COVID-19.

Due to the severity that some COVID-19 cases progress to, hospitalization is required, and these cases may progress to ICU admission. This inflicts enormous stress on healthcare workers as hospitals are working at full capacity and at times lack of sufficient equipment. The occurrence of hospitals frequently reaching high or full capacity is becoming an overwhelming and alarming issue, as noted by the CDC's COVID-19 Module Data Dashboard (2). This results in extensive physician burnout (3) which can be detrimental to physician-patient interaction.

This stress can be alleviated with a more succinct understanding of which individuals are at an increased risk of fatality caused by COVID-19. Therefore, it would be beneficial to identify patients who merit priority and order treatment accordingly to these high-risk cases. Priority treatment would be given to patients who have a high likelihood of a fatal outcome, given their present state when tested positive for the virus. The limited resources housed by hospitals can be more appropriately allocated and there would be a decrease in the number of hospitalized patients under the care of overworked hospital employees.

Deaths that are caused directly by COVID-19 infection are not the only ones that should be discussed. There is an abundance of evidence suggesting intrahospital transmission of the virus. This transmission has occurred to both hospital patients, who may already be immunocompromised, and staff working tirelessly to save lives. Gold et al. noted more than 5,000 cases of this type of transmission occurring between May 14 and June 21 (4). With an increased number of COVID-19 patients admitted to a hospital, there is a higher probability of COVID-19 becoming a nosocomial disease for some inpatients. Moreover, patients with chronic or acute illnesses may have had to delay or cancel their hospital treatment appointments, due to hospitals reaching their capacity (5). As a result, their non-covid disease may have progressed to a severe point, or even death. With increased consideration taken into account when admitting patients, the persistence of these avoidable deaths will decrease.

However, it is difficult to predict the high fatality risk of a patient who should be admitted to a hospital with high priority since there are a myriad of different factors that contribute to an individual's infection progression once they test positive for covid-19. All of these metrics may be diverse, leading physicians to a disruptive confusion as to which factors to rely on, especially when each patient is unique. Complete knowledge into how this virus manifests itself in the body is lacking. Each case of COVID-19 contains distinct epidemiological features that have the power to dictate the progression of the disease and whether the outcome will result in death. There is a vital need to be able to identify these factors in order to prioritize care to those who are at greatest risk and prevent future death with this increased and proactive care. A single algorithm, like the one produced in this study, that combines all of an individual's dispositions to make a prediction would therefore be useful. This solution to improve and prioritize hospitalization with our fatality prediction approach will be able to alleviate the burden of hospitals reaching capacity, reduce medical worker burnout, and minimize the unintentional spread of the virus. Furthermore, the ability to identify and prioritize serious cases which may result in death might be life-saving for critical patients. All of these factors will benefit the COVID-19 infected individual, the healthcare employees, and hospital inpatients.

It is imperative to be able to predict which individuals tested positive for COVID-19 should be hospitalized for immediate care. This study aims to create a prediction model to be able to correctly identify patients who are at an increased risk of death, following a COVID-19 diagnosis. Utilizing informed decisions with our predictor, alongside medical expertise from medical professionals at the scene, physicians can determine with greater certainty which individuals should receive hospitalization.

## Materials and Methods

### Data Source

In this study, two publicly available epidemiological datasets were obtained, processed, and used for analysis. To monitor and anticipate spread of virus during the COVID-19 outbreak, a real-time database of individual-level epidemiological data is collected and published on GitHub (https://github.com/beoutbreakprepared/nCoV2019) (6). The dataset is supplied from an open working group repository to promote and enable the sharing of public health data to advance the field of public health. It incorporates data from a number of different sources to provide individual-level data instead of aggregate data provided by most data repositories.

Each case in the database represents an individual tested positive for COVID-19, gathered from different sources. This dataset originally contains 2,310,111 cases. To protect the privacy of patients, each case is deindividualized and anonymized. The cases are labeled with an “ID” noted in the dataset, which is only used to keep track of cases and has no relation to the actual individual. This file contains the variables including ID, age, sex, city, province, country, latitude, longitude, date onset, date admission, date confirmation, etc. Each variable is described below and this dataset will be referred to throughout the paper as the GitHub dataset.

**ID**—unique label for each deindividualized case
**Age**—age at time of positive covid-19 test
**Sex**—sex of the case
**City**—geographic location of case
**Province**—first administrative division where the case is reported
**Latitude**—latitude where case is reported
**Longitude**—longitude where case is reported
**Date onset symptoms—**date when the case began exhibiting symptoms, if symptomatic
**Date admission**—date when the case is reported to have been hospitalized
**Date confirmation—**date when the case is reported to have been tested positive for COVID-19, by a rt-PCR test
**Symptoms**—symptoms recorded for the case
**Lives in Wuhan**—“yes” if case is resident of Wuhan, “no” if case is not resident of Wuhan
**Travel history**—travel dates to and from Wuhan that were recorded for the case
**Reported market exposure**—“yes” if market exposure was recorded, “no” if it was not
**Additional information**—extra information that is informative about the case
**Chronic disease binary**-−0 entered for a case without chronic disease, 1 entered for a case with chronic disease
**Chronic disease**—listed specific chronic disease per case
**Source**—URL of origin of information for each case
**Outcome**—“died” or “discharged” from hospital
**Date of death or discharge**—date of death or discharge that was reported

A smaller dataset that contains additional and extensive detailed information on the predisposition of the patient was then used. This dataset was obtained from https://datarepository.wolframcloud.com/resources/Patient-Medical-Data-for-Novel-Coronavirus-COVID-19. It includes patient medical data for those tested positive for coronavirus that was made to be computable. Here, a larger ratio of records contain specific symptoms and chronic diseases of applicable cases are included in a detailed account, compared to the previous GitHub dataset. As of June 30, this dataset contained 217,192 cases. This dataset will be referred to throughout the paper as the Wolfram data set. Wolfram data set includes variables as below:

**Age**—age of case at time of positive covid-19 test result
**Sex**—reported gender of case
**Date of onset symptoms**—initial date of reported symptoms
**Symptoms**—specific symptoms exhibited by case
**Travel history**—travel locations of case
**Chronic diseases**—specific chronic diseases of case
**Date of discharge or death**—recorded date of death or discharge of patient
**City**—city of residence for positive case
**Administrative division**—City, country for positive case test administration
**Country**—country of residence for case
**GeoPosition**—geographical location of case
**DateOfAdmissionHospital**—Date case was admitted to hospital, if applicable
**DateOfConfirmation**—Date case was confirmed to be positive for covid-19
**LivesInWuhan**—“True” if individual lives in Wuhan
**TravelHistoryDates**—Dates of travel history for the case, if applicable
**TravelHistoryLocation**—If applicable, lists location infected individual traveled
**ReportedMarketExposure**—Reported as “Missing,” “True,” or “False”
**ReportedMarketExposureComment**—Details where public market exposure to the virus occurred for the case
**ChronicDiseaseQ**—“True” if individual has chronic disease, “False” if no
**SequenceAvailable**—“True” is available
**DischargedQ**—if individual was discharged from hospital
**DeathQ**—if outcome was death for individual
**DateOfDeath**—date of death in DD.MM.YYYY format if individual died
**DateOfDischarge**—date of discharge in DD.MM.YYYY format if individual was discharged from hospital

There is a tradeoff between the quality of these two datasets and by using them both, we hoped to reconcile this circumstance. The GitHub based dataset used was advantageous due to its large size. Unfortunately, it lacked precise information on the specific symptoms and chronic diseases each case faced. The Wolfram dataset was beneficial due to its multiple attributes that include specific symptoms and chronic diseases. Both datasets were used separately to train the machine learning models in order to reflect the prediction capability of datasets of different qualities.

### Methodology

#### Data Processing

The GitHub data was preprocessed to keep the variables age, sex, latitude, longitude, symptoms, chronic disease, outcome, and travel history. Other variables included in the original dataset that were removed for analysis consisted of additional written information relating to the case and its report. Much of this was left empty and did not pertain to the information valuable to predicting death. Symptoms and chronic diseases are converted to a binary variable, indicating a patient has COVID-19 symptom/chronic disease or not. A new column was created, titled “combined symptoms.” If the individual had at least one symptom of any kind, there was a 1. The same is true for chronic diseases. The binary converter was utilized due to the lack of specific information regarding the symptoms and chronic diseases each case possessed. Data was limited to merely knowing if an individual harbored symptoms and/or chronic diseases, but not which ones. Outcome was either “death” as a 1 or “alive” as a 0 in the new “death” column. The GitHub data originally had over 3 million records. Cases with missing data were removed from the set and 28,958 cases were left. There were 530 deaths in the data after pre-processing, indicating a 1.83% death rate.

The Wolfram dataset included specific information on the clinical history of some patients and the symptoms exhibited. Unlike the Github dataset where we merely used the variables of “presence of symptoms” and “history of chronic illnesses” for analysis, we were able to specify and categorize the symptoms and comorbidities included. This is advantageous as it allows for detailed information to be geared toward unique individuals and cases with differing medical histories and symptoms present. Missing values were removed from the dataset and the final dataset resulted in 1,448 records with 123 deaths cases, indicating a 8.5% death rate. Symptoms and chronic diseases were then grouped into categories. We found 114 unique symptoms listed in the original dataset, which were divided into the following categories: “respiratory,” “weakness/pain,” “fever,” “high fever,” “gastrointestinal,” “nausea,” “cardiac,” “kidney,” and “asymptomatic,” and “other.” High fever was noted if the fever temperature recorded is above 39 degrees Celsius. Forty-seven unique chronic diseases were categorized into “diabetes,” “neuro,” “hypertension,” “cancer,” “ortho,” “respiratory,” “cardiac,” “gastrointestinal,” “kidney,” “blood,” “prostate,” “thyroid,” and “none.” If a patient exhibited a symptom or chronic disease, a 1 was inputted in the corresponding column. This dataset was filtered to create one that only includes these unique symptoms and chronic diseases, age, gender, and death. Age was put into age ranges of intervals of 10 years from 0 to 99 years old.

For both datasets (Table 1), data was split into train, validation, and test groups. Thirty percent of the data was included in the test group. From the remaining data, 70% was assigned as the training data and 30% was included as the validation data. Once the data was properly separated, machine learning models discussed below were applied to do prediction, and the validity and prediction power of these algorithms were assessed with various metrics.

**Table 1:** Comparison of the attributes included in the two filtered datasets.

**Attribute**	**Description**	**G**	**W**	**Attribute**	**Description**	**G**	**W**
ID	ID issued to each deindividualized case in the dataset	✓	✓	High fever	0—individual did not have a high fever (>39*C) 1—individual had a high fever		✓
Age range	Age range individual's age falls into during time of positive COVID-19 test	✓	✓	Kidney S	0—individual did not display kidney related symptoms 1—individual displayed kidney related symptoms		✓
Gender	Reported gender of individual	✓	✓	Asymptomatic	0—individual is displaying symptoms 1—individual does not show an symptoms		✓
Latitude	Latitude where case is reported	✓		Diabetes	0—individual does not have diabetes 1—individual does have diabetes		✓
Longitude	Longitude where case is reported	✓		Neuro	0—individual does not have neurological chronic disease. 1—individual does have neurological chronic disease		✓
Symptoms	0—individual displayed no signs of symptoms 1—individual displayed signs of symptoms	✓		No chronic Disease	0—individual has chronic disease history 1—individual has no history of chronic disease		✓
Chronic Disease	0—individual had no reported chronic disease history 1—individual had history of chronic disease	✓	✓	Hypertension	0—individual does not have hypertension 1—individual does have hypertension		✓
Outcome	0—alive 1—dead	✓	✓	Cancer	0—individual does not have cancer 1—individual does have cancer		✓
Respiratory S	0—individual did not display respiratory symptoms 1—individual displayed respiratory symptoms		✓	Orthopedic CD	0—individual does not have orthopedic related chronic disease 1—individual does have orthopedic related chronic disease		✓
weakness/pain	0—individual had no weakness or pain 1—individual felt weakness or pain		✓	Respiratory related CD	0—individual does not have respiratory related chronic disease 1—individual does have respiratory related chronic disease		✓
Low fever	0—individual did not have a low fever (<39*C) 1—individual had a low fever		✓	Cardiac related CD	0—individual does not have cardiac related chronic disease 1—individual does have cardiac related chronic disease		✓
Gastrointestinal S	0—individual did not display gastrointestinal symptoms 1—individual displayed gastrointestinal symptoms		✓	Kidney related CD	0—individual does not have kidney related chronic disease 1—individual does have kidney related chronic disease		✓
Other symptoms	0—individual did not display other 1—individual displayed other symptoms		✓	Blood related CD	0—individual does not have blood related chronic disease 1—individual does have blood related chronic disease		✓
Nausea	0—individual did not experience nausea 1—individual experienced nausea		✓	Prostate related CD	0—individual does not have prostate related chronic disease 1—individual does have prostate related chronic disease		✓
Cardiac S	0—individual did not display cardiac related symptoms 1—individual displayed cardiac related symptoms		✓	Thyroid related CD	0—individual does not have thyroid related chronic disease 1—individual does have thyroid related chronic disease		✓

#### Autoencoder for Rare Event Detection

**Figure 1:**
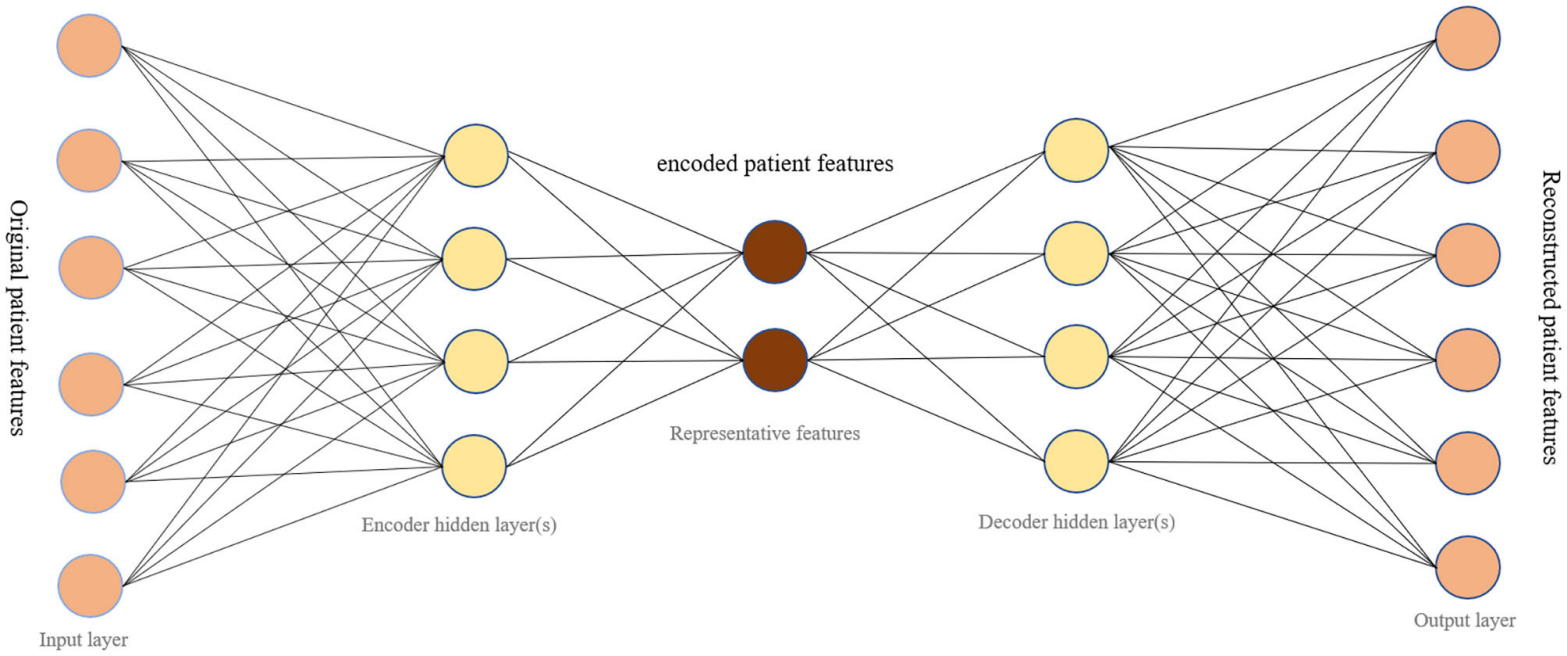
The network structure of an autoencoder.

Typically, an autoencoder is a neural network that learns representative codes from input and maps these codes back to the input (7). This model is generally used to encode input variables and output a compressed version of that input. It consists of two main parts: the encoder and the decoder (Figure 1). The encoder is the part that learns about the deep features of input data. The decoder relies on learnt features to recreate the original data provided. There are three layers to the autoencoder model used: input, output, and hidden. In our research, the input layer of the network is a vector recording patient information. Hidden layers in the encoder learn a small vector representing input data. The decoder then maps hidden layers to a vector with the same dimension as the original input vector. The goal of training an autoencoder is to minimize the mean square error between the input vector and the reconstructed output vector, while also avoiding overfitting the data.

With the capability of learning representative features for input dataset and reconstructing data from extracted features, the autoencoder model can offer a solution for anomaly detection when it is trained on normal dataset (8). During the training process, the model ingests a series of normal data and learns latent common features of all normal dataset. When the trained model encodes and decodes an anomaly dataset, the reconstruction error is usually large since the model only learns how to reconstruct normal data. This means that an input data can be considered as an anomaly when the model reconstructs it with a high reconstruction error if the model is pre-trained with normal data. Similarly, the autoencoder can serve as a rare event prediction solution, in which the autoencoder is initially trained on majority events related data, the data that is considered to be normal.

Our study relied on the latent function of autoencoders, anomaly detection, to create a model that would predict fatality upon one's COVID-19 diagnosis. Death from COVID-19 constitutes an anomaly because of how infrequently it occurs in our dataset. The autoencoder has been transformed from a data compression algorithm, to a rare event prediction model with the ability to distinguish between life and death. In other words, the original classification problem in our study was transformed into an anomaly detection problem.

**Figure 2:**
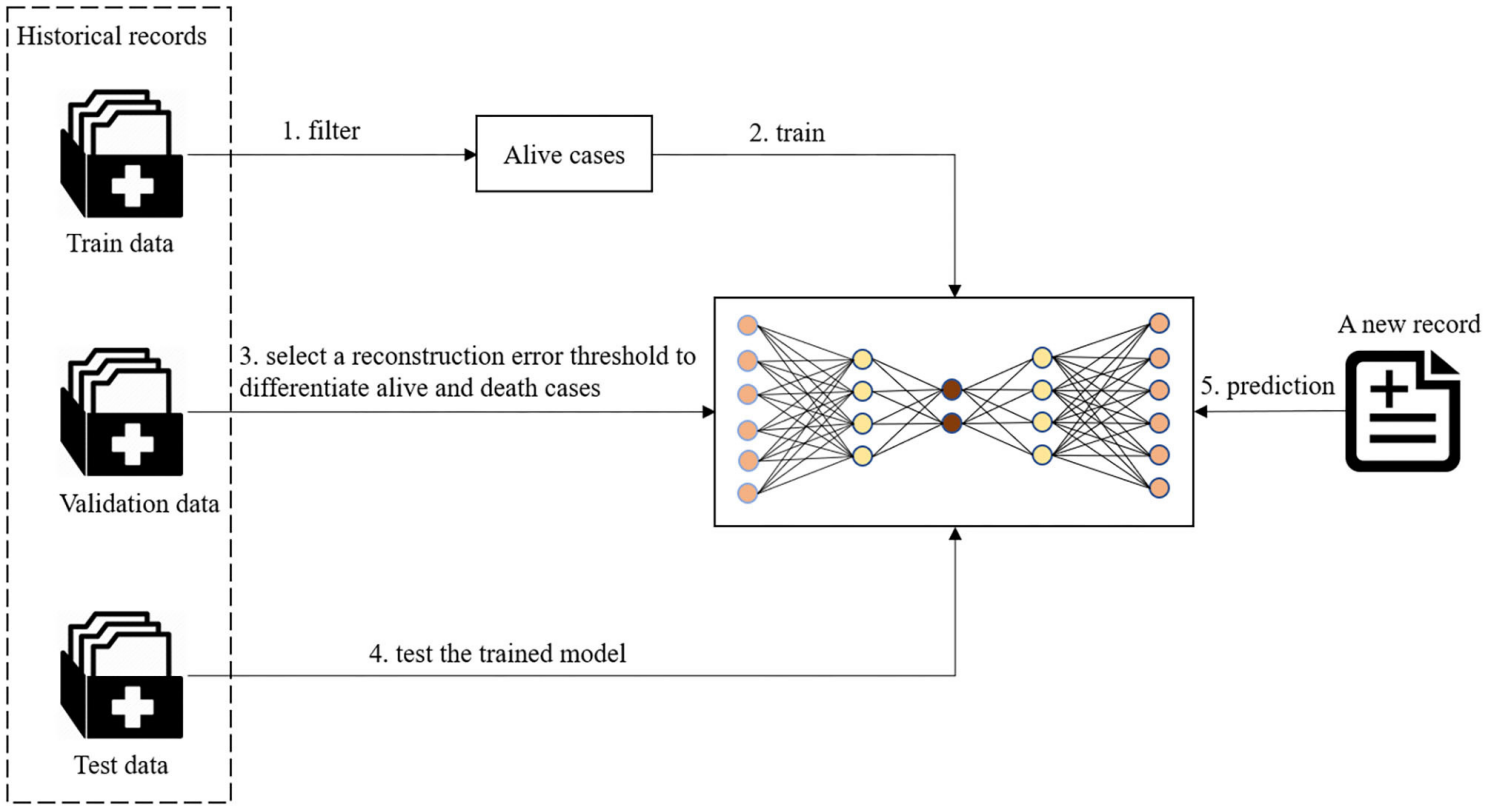
The workflow of fatality prediction.

As shown in Figure 2, the preprocessed data was split into train, validation, and test groups. For training the autoencoder model, a subset of data in the train group was used that solely contained non-death cases. The data was transformed to fit a standardized Gaussian curve before being input into the autoencoder model. The autoencoder model learns representative features of alive patients from their demographic information, symptoms, and chronic disease illnesses. After an autoencoder model was trained on individuals who tested positive for COVID-19 and survived in the train dataset, a series of reconstruction errors was populated by applying the trained model on validation data with different thresholds. The reconstruction error which performed best at differentiating the living and dead patients was selected as the threshold to determine a death prediction for a patient. Different thresholds were tested to find which would allow for the most optimal trade-off between recall and precision. Classification followed suit, where high construction errors were noted as a rare event. In this study, this would pertain to death.

### Evaluation

For each of the datasets we used, the Github dataset and the Wolfram dataset, an autoencoder model was trained to predict fatality in the test dataset. For comparison, the train data set was also trained with logistic regression, random forest, support vector machine (SVM), SVM one class models, isolation forest, and local outlier factor. Logistic regression, SVM, and Random forest are three widely used classification methods. A logistic regression model predicts the probability of a categorical dependent variable occurring. The SVM model seeks to find the hyperplane that has a maximal distance between two classes. Random forest is a tree-based learning algorithm which utilizes decision trees rising from the training subset which are selected randomly to solve a classification problem. The One-class SVM algorithm is usually adopted for Novelty Detection, determining whether or not a new data record is similar to the training set, which only contains “normal” data. Both isolation forest model and local outlier factor model are outlier detection algorithms; the former works by explicitly isolating points that deviate in the dataset and the latter uses the density surrounding data points to determine whether or not they are outliers.

In order to detect which model has the best predictive power, these models were evaluated with multiple metrics, including accuracy, specificity, sensitivity and the area under the curve (AUC) score.

#### Accuracy

Accuracy presents how many predictions the model has gotten correct. For this project, this would equate to the number of correctly predicted deaths and survivals over the total predictions made by the model. Unfortunately, since our data is highly imbalanced, accuracy is not a reliable metric. Our dataset contains a large amount of survival cases, causing a skew in that our models would predict “alive” a lot more often than “death,” which leads to a high accuracy regardless if the “death” predictions are accurate, since there are so few. It is the fraction of correct predictions:

$$\begin{array}{l} Accuracy=\\ \frac{True\;Positive\;+\;True\;Negative\;}{\;True\;Positive\;+\;False\;Negative\;+\;False\;Positive\;+\;TrueÑegative} \end{array}$$

#### Specificity

Specificity is the rate of true negatives. It measures the proportion of true negatives that the model accurately predicts as negative. In this study, a true negative would be a prediction of “no death,” when the individual did not die. Specificity is less important of a metric than sensitivity, in our study, because it is more important to identify those individuals who have a greater likelihood of death, rather those who do not. Therefore, in order to ensure that we do not forget to account for any individual who can potentially encounter death, we can risk having some false positives of individuals who will receive care regardless if their prediction of death is accurate or not. It is measured by:

$$\begin{array}{l} Specificity=\frac{True\;Negative}{False\;Positive\;+\;TrueÑegative} \end{array}$$

#### Sensitivity

Sensitivity is the rate of true positives. It measures the proportion of true positives that the model predicts accurately as positive. A model with high sensitivity when dealing with an outcome of fatality is ideal. For this study, a true positive would be a prediction of death that is accurate. It is calculated by:

$$\begin{array}{l} Sensitivity=\frac{True\;Positive}{True\;Positive\;+\;FalseÑegative} \end{array}$$

#### AUC

AUC stands for “area under the curve.” In order to detect which model had the best predictive power, we calculated their AUC value. The curve being referred to is the ROC curve, which contains the sensitivity metric on the y-axis and specificity on the x-axis. Since there is always a trade off between these two metrics, an ROC curve best displays their interaction in the model used. A curve which depicts a model that has a high value for both will be close to a 90° angle. An AUC value for a desired curve like this is 1, the maximum. An ROC curve that is no better than a random guess will be a line with a slope of one and have an AUC of 0.5.

## Results

### Results on the Github Dataset

**Figure 3:**
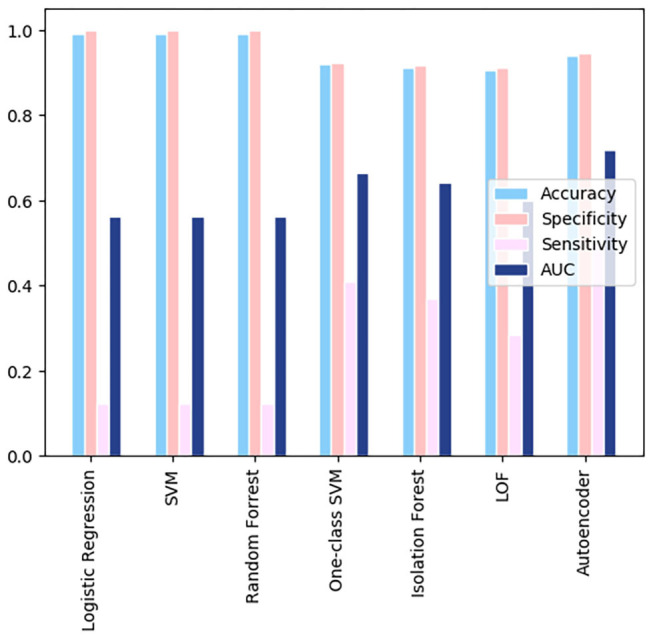
A comparison of the different models using the GitHub data.

A comparison of the different models using the GitHub database is shown in Figure 3. All the models shown resulted in specificity and accuracy values above 0.9. Logistic regression, SVM, and random forest all have sensitivities below 0.4. Autoencoder scores above a sensitivity value of 0.4, leading it to have the best sensitivity. AUC scores, which are dependent on both the sensitivity and specificity of the models, are highest for the autoencoder model. The AUC scores for the remaining three models are almost identical, due to their similar specificities and lower sensitivities. The overall best results were obtained from the autoencoder model. The low metric values are results of the very few instances where health related attributes are included in the Github dataset, leading to a generalized analysis.

### Results on the Wolfram Dataset

**Figure 4:**
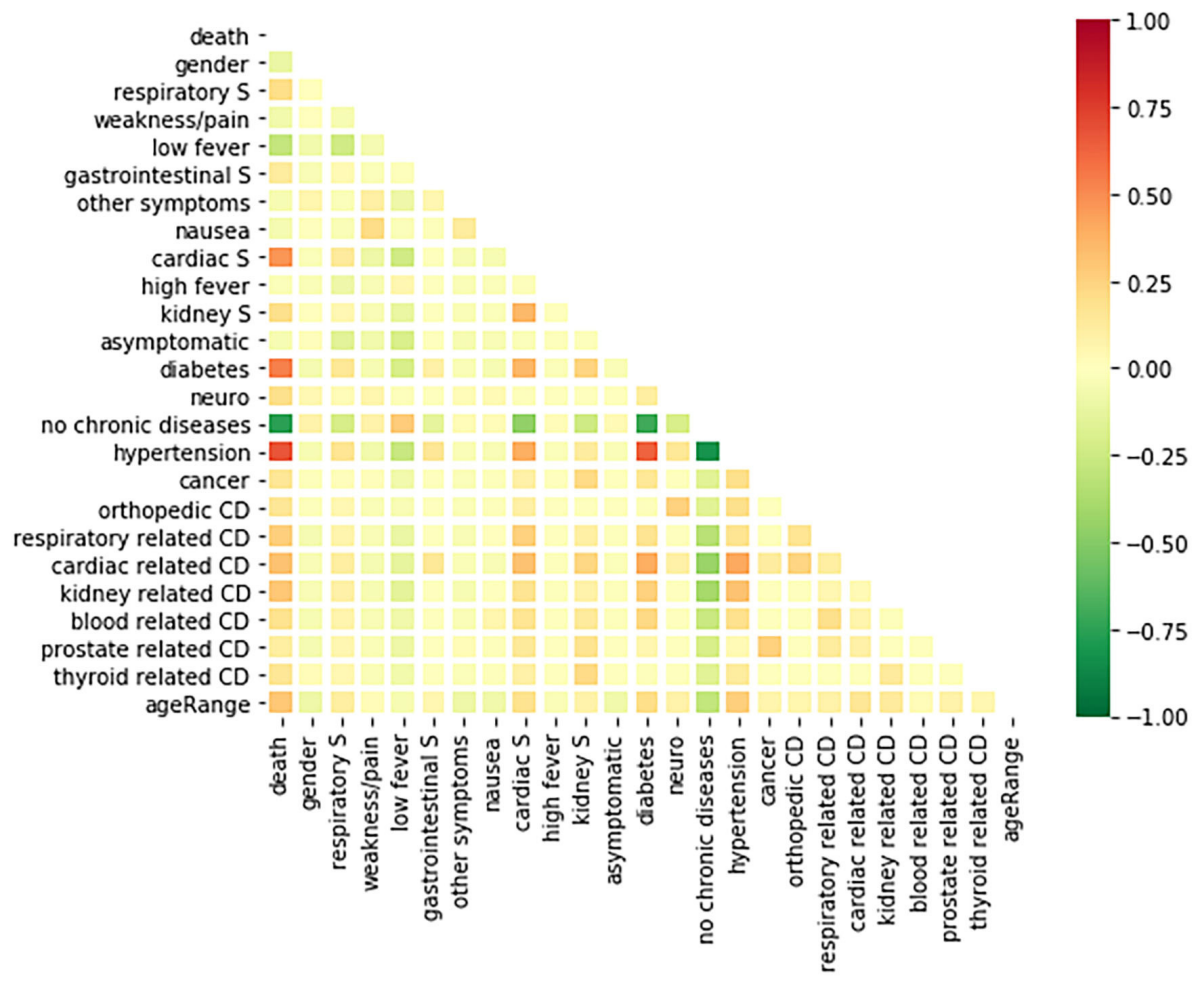
A correlation matrix of the variables used in the analysis of the Wolfram database.

For the Wolfram dataset, a correlation matrix depicts the relationships between all variables analyzed in our models. The correlation matrix is shown in Figure 4. A deeper red color indicates a more positive linear correlation and a deeper green color indicates a more negative linear correlation between the two variables in question. It can be seen that the square at the intersection between “no chronic diseases” and “death” represents the strongest negative correlation. The matrix displays a strong correlation between fatality and COVID-19 patients with chronic diseases.

**Figure 5:**
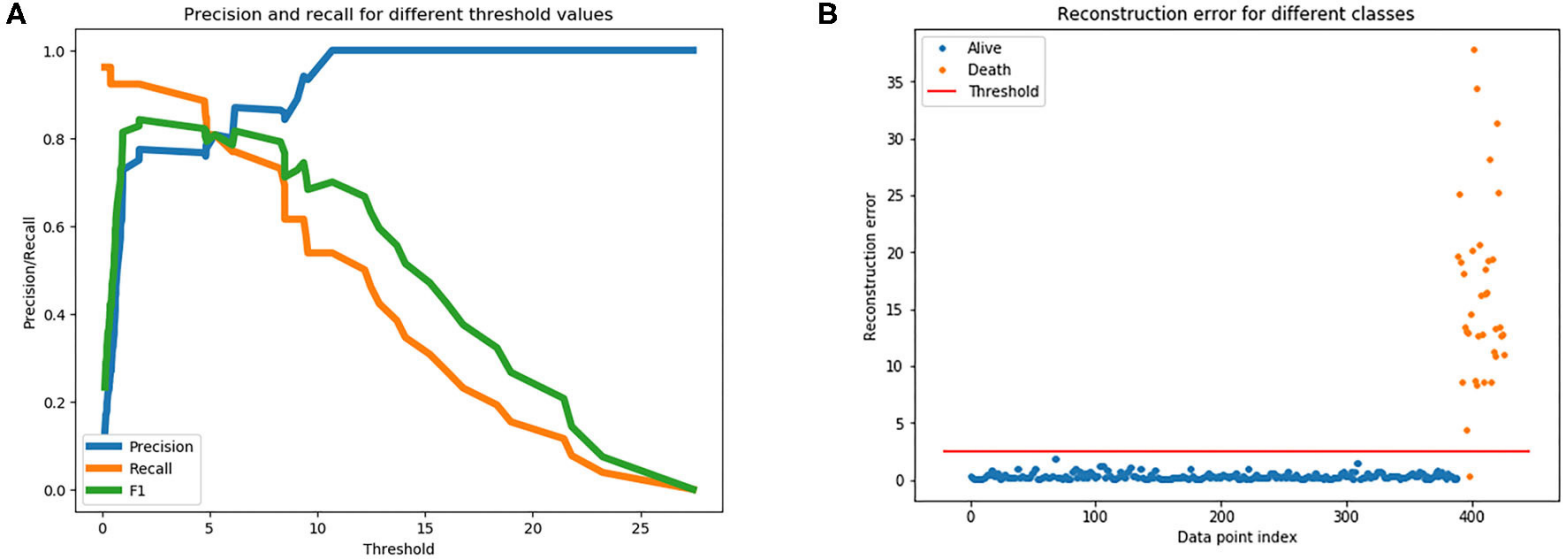
**(A)** Is used to gather a threshold for the autoencoder model. **(B)** The reconstruction error at the chosen threshold of 2.5 on validation dataset.

To train an anomaly detection model on the Wolfram dataset, the autoencoder model learned high-level features from all survival cases from the train data set, and all cases in the validation dataset aid the selection of a threshold to differentiate survival and death cases. Figure 5A is a plot of the various precision, recall, and F1 scores across different thresholds when predicting fatality on the validation dataset using the trained autoencoder model. The plot supported determining which threshold would be an optimal choice. It was decided, upon examining this plot, that a threshold of 2.5 could be used for our dataset. Figure 5B graphs the reconstruction error of all cases in the validation dataset at the chosen threshold. The dots above the threshold line show the true positives and false positive prediction cases. Orange dots represent death outcome and blue dots represent survival outcome. Only orange dots appear above the threshold line, signaling that there are only true positives present above the threshold.

**Figure 6:**
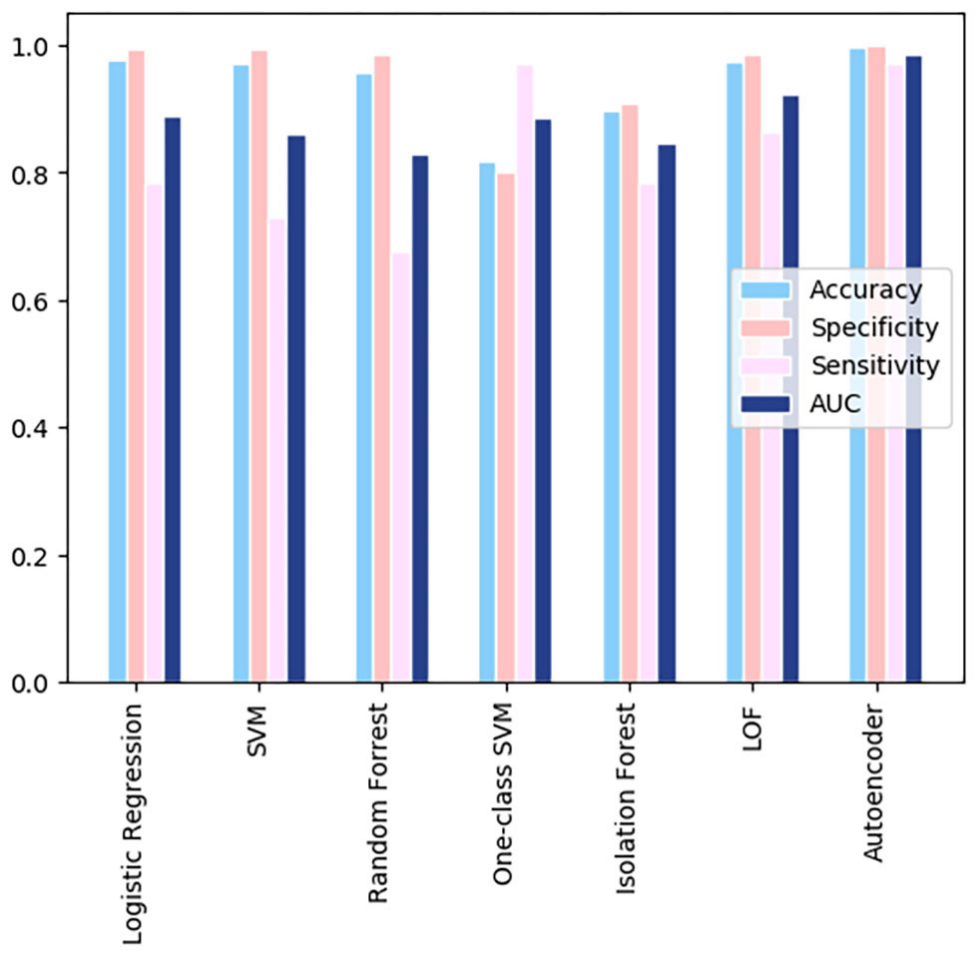
A comparison of the resulting metrics of 7 models used in the Wolfram dataset.

When calculating the results of the autoencoder model using our selected threshold value, 36 out of 37 deaths in the test dataset were correctly predicted by the model, resulting in a 97% sensitivity rate. Lastly, the metrics across the various models for the Wolfram dataset were compared, which can be visualized in Figure 6. As shown in the figure, autoencoder is the optimal model as its results are highest in every metric depicted. There is vast improvement in these models when fed the Wolfram data vs. the GitHub data because the wolfram dataset contains detailed health related information. One-class SVM, isolation forest, local outlier factor, and autoencoder account for anomalies in the data, leading to their high sensitivity values. However, there is a tradeoff between one-class SVM's high AUC and sensitivity with its lower accuracy and specificity.

## Discussion

### Related Works

#### Machine Learning in Studying COVID-19

Machine learning has found its niche in the medical field. With the emergence of new medical data constantly created, these algorithms can serve as classification for the purpose of diagnosing a slew of diseases. One particular instance of this was done by researchers at Harvard Medical School (9). Using data from a cancer registry, they were able to make a super learner prediction function to classify the current stage of lung cancer progression in a patient.

Machine learning algorithms have been a prominent force in studying COVID-19. One piece of information researchers are sure about is the fact that it is beneficial to diagnose a COVID-19 infection earlier than later. Since it is primarily a respiratory infection, a study (10) looked at CT scans for COVID-19 classification. Using an SVM model, they were able to find features of these images that are specific to COVID-19 infections in order to classify the disease. This method achieved a 98% accuracy score at classification.

Transitioning from diagnosing COVID-19, some studies focused on identifying regional death patterns relating to the pandemic. One specific study predicted short term (7 days) fatality at the county level (11). In the study, they collected 23 different datasets, ranging from overall country level deaths to hospital data, and used five different predictors to determine COVID-19 death count for county wide visualizations. These predicted models can be used to determine which hospitals should be prioritized to receive supply and how limited resources should be distributed nationwide. Attempting to study factors outside of an individual's control, a study looked at temperature (12) and COVID-19 death patterns in different areas, aiding hospitals in deciphering disease patterns. However, with the minor association between death and temperature, more variables should be included into the fatality risk model.

Currently, many studies are emerging that utilize machine learning algorithms to predict the death risk of a patient who has tested positive for COVID-19. Most of these studies have access to hospital medical records of the patients and analyze all corresponding information including demographic, symptoms, comorbidities, lab results, medical images. One such study utilized five machine learning approaches (logistic regression, partial least squares regression, elastic net, random forest, and bagged flexible discriminant analysis) to determine which factors are most associated with an individual's prognosis (13). These factors were combined to create a mortality risk score to estimate the mortality risk for 183 individual patients. Age, high-sensitivity C-reactive protein level, lymphocyte count, and d-dimer level of COVID-19 patients at admission are the factors concluded to be associated with increased mortality risk in the study. Another similar study attempted to create a clinical score to identify patients who had an increased risk of serious disease progression (14). Medical chart information of admitted patients were collected and variables correlated to critical illness with *p* < 0.02 selected to create an online calculator for the likelihood (with 95% CIs) that a hospitalized patient with COVID-19 will develop critical illness. Logistic regression and separately LASSO regression were used to determine 10 variables that served as a significant predictor of a severe and critical COVID-19 infection. Nemati et al. analyze survival characteristics of a group of 1,182 patients to test different variables of a patient and their overall survival to aid public health officials in their decisions regarding COVID-19 outbreaks (15). They discovered that gender and age were the two largest contributing factors to fatality, e.g., men had a higher fatality rate than women, agreeing with an original sample found in China early on (16). They also had looked for correlations underlying a patient's discharge time under COVID-19. By evaluating discharge times of individual patients with different machine learning methods, the researchers found that the gradient boosting survival analysis model was superior to other methods.

These individual level fatality studies are limited in predicting hospitalization due to their efforts to identify severe risk in patients after they are already admitted to a hospital. Most of the variables found to have a correlation with COVID-19 related deaths requiring intensive care is based on inpatient hospital lab results. These patients are already in the hospital, taking up capacity and resources. However, if fatality prediction can be developed that relies primarily on outside information, this could prove beneficial in allowing full hospitals to make informed and rational decisions on who to admit when the disease is not currently life threatening (e.g., when the patient is only experiencing minor symptoms). This will alleviate physician stress, reduce risk of virus spreading throughout the hospital, and conserve hospital resources. The proposed study will focus on the individual level detailing of the fatality risk based on their unique demographics, symptoms, and comorbidities using machine learning methods.

#### Rare Event Prediction

The mortality rate for COVID-19 is a difficult calculation due to the number of people who may be infected but show no symptoms, and therefore proceed undetected, and the lag time between infection and death. Following extensive discussion from epidemiologists and scientists, the current consensus is that the fatality rate ranges between 0.5 and 1% (17). However, this percentage is dependent upon location, patient demographics, and physician experience in treating COVID-19. In a machine learning perspective, this rate constitutes a death event as a rare event.

Rare events are events that occur infrequently, such as major earthquakes, hurricanes, floods, asteroid impacts, forest fires, financial market crashes, epidemic disease spread. “Rare events are often interesting events” (18) since these events can have extensive effects that have the potential to rupture the equilibrium of systems such as the stock market and society in general. Rare event detection/prediction would be beneficial for the community to be able to better prepare for events which occur at low frequency, but lead to huge loss. However, rare event detection/prediction is a difficult task because the occurrence of a rare event is usually <5% of all events, comprising a small percentage of data. In most instances dealing with rare events, insufficient data is gathered for a thorough analysis.

Researchers are becoming increasingly interested in using machine learning methods to detect/predict rare events using classification models or anomaly detection models. For classification models, a majority of machine learning models assume that the dataset is balanced and make predictions based upon those assumptions. A significant issue with rare events classification is that accuracy is not a reliable metric to evaluate the model because the desired event occurs too infrequently. A very high accuracy can be achieved when all events are classified as non-rare events. In medical fields, Luca et al. used a model based on Extreme Value Theory (EVT) to predict epileptic seizures for people using electroencephalogram (EEG) devices (19). They focused on hypermotor seizures that are located in the tails of a normal, standardized curve for normal movement behavior. The percent of epileptic movements per patient has a mean of 2%. This rarity of events does not allow for sufficient training data and results in an imbalanced dataset. It was noted that the datasets with the greatest amount of rare events have the EVT model result in higher sensitivity than the standard SVM machine learning model. Additionally, Sarker et al. used data from social media and search engines to predict and monitor adverse drug reactions (ADR) (20). They find the use of social media in monitoring ADRs are increasing as methods are becoming increasingly accurate and the detection time is significantly lower compared to traditional detection methods. Conner et al. similarly uses a Twitter corpus of symptom-related Tweets to detect and predict ADRs (21). By using keywords, they eliminate many irrelevant Tweets and categorically find tweets that strictly relate to the discussion of a drug and its symptoms. This study was furthered improved in the detection of adverse effects from vaccines (22). Using Multi-instance Domain Adaptation (MIDA) model on Twitter data, they were able to identify symptoms and align them with formal reports of symptoms to identify adverse effects in the use of a vaccine. Yates and Goharian also attempted to detect expected and unexpected ADRs by mining drug review sites for symptoms contained in the Unified Medical Language System (UMLS) (23). These methods could be applied across the world, as (24) shows that the same methods work in Spanish. His team scanned Twitter, Facebook, and Spanish medical forums using symptom related keywords and were able to detect symptoms.

Deep learning, overall, utilizes multiple layers neuronal networks to withdraw higher level features from the simpler input, emerging as an innovative way to improve the performance of data-driven applications recently. Many researchers have begun to use deep learning to solve rare event detection and prediction issues. A previous study used deep learning methods to predict the rapid intensification of a tropical cyclone (TC), a rare event in natural disasters, using a plethora of factors that contribute to a TC's intensity (25). This can prove critical in saving lives from disastrous situations. A study compared traditional machine learning models to recurrent neural network (RNN) to predict early signs of heart failure and delay one's progression to receiving that diagnosis (26). This was complicated in the past with classical machine learning models because of the intricacy of electronic health records and all of the information involved to make these predictions. This paper conveys the revolutionary pathway being carved out by deep learning methods in medicine. Not only can deep learning solve the issue of rare event detections, but it can also be applied to more discrete problems that medical researchers face. Wang et al. (27) was able to identify potential adversarial effects through a multi-instance logistic regression model (MILR) by scanning Tweets and using VAERS information. Wang et al. (22) also uses deep learning with sSSM and nSSM models to classify the discussion of symptoms within tweets that relate to the flu. Symptoms such as arm pain and headaches could be identified in tweets related to the flu accurately under their model. In our research, we will use deep learning methods to do the fatality prediction and compare their performance with traditional machine learning methods.

### Findings of Our Study

A series of machine learning models were compared to validate which one worked best in this scenario of death prediction. Two different datasets were used: one that specified symptoms and comorbidities and one that generalized across them. Fatality related to COVID-19 is caused by a multitude of different, confounding factors. Therefore, introducing a model that can predict whether an individual's diagnosis of COVID-19 is likely to be fatal will serve as a reliable and advantageous tool. Every case of COVID-19 is unique, and this predictor model accounts for the significant features present in a COVID-19 diagnosis and relies on information that does not require any hospitalization. It focuses on demographic information, symptoms, and patient chronic disease illness. Therefore, it can be used to make a fatality prediction prior to hospitalization of the infected individual. This will provide guidance for employees at hospitals that are reaching or at capacity to make educated decisions upon whether or not to admit a patient. Cases of COVID-19 continue to rapidly rise at alarming rates, reported by CNN (28), and it is projected that the virus will persist for a continued amount of time. It is crucial that patients who are at an increased risk, where death is imminent, receive care in hospitals to prevent this outcome.

The GitHub dataset was largely generalized; it did not include specifics on symptoms or chronic diseases that individuals harvested. Moreover, death made up a very small percentage of the cases in our dataset. The results of different machine learning models indicated that the autoencoder, a deep learning model, produced the best prediction results. However, no model produced a sensitivity metric above 0.5, showing limitations of the model. The sensitivity metric is the most important as we want to minimize the number of false positives in order to avoid missing a patient who is in danger of a death outcome.

The Wolfram dataset incorporated specific symptoms and chronic diseases that plagued the individuals in the data. This allows for models based on this data to be a better fit and cater to the unique characteristics of the individuals who have the virus. Similarly, the autoencoder proved to have the most optimal results upon comparison with all of its metrics being the highest.

COVID-19 has an average fatality rate between 0.5 and 1% (17). Although this is a high fatality rate for a virus, death is still considered as a rare event for machine learning algorithms to learn. Autoencoder serves as the best prediction model for COVID-19 death in our experiments when it converts the prediction problem to an anomaly detection problem. The results demonstrate that it will serve as the most representative model for physicians to be able to make an educated decision as to whether or not to admit a patient to a hospital and decide how extensive treatment should be a valuable tool in making decisions for the best course of action of care. However, it should not be the only resource used to make this decision. The healthcare worker will also take into account additional knowledge on the patient and use their best judgement, along with the information provided by the model.

The correlation matrix produced by the Wolfram dataset provides insight into which factors are most notable in their association with a patient's fatality. There is a high correlation between death and having a chronic disease. This is displayed by the result that having no chronic disease history and death occurring share a strong negative correlation based on the matrix. More specifically, if the individual has a chronic disease of hypertension or diabetes, they have a higher chance of death than other comorbidities in the study. Turning our attention toward symptoms, gastrointestinal, kidney related, and respiratory symptoms are shown to be positively correlated with death. Cardiac related symptoms seem to be the symptoms, out of the ones studied here, that are most correlated with COVID-19 death (29). The r value between having cardiac symptoms and death occurring is between 0.5 and 0.75, displaying a moderate positive correlation. The CDC similarly reports that individuals who have underlying chronic diseases report greater chances of hospitalization and death (30). This information shows that individuals who have chronic diseases should take greater precaution toward both reducing their risk of contracting the disease and receiving care if infected. Interestingly, having a low fever (below <39°C) has a weak negative correlation with death. This agrees with current data knowledge that states a low fever is a common symptom for a mild COVID-19 case (31).

### Limitations

There are limitations embedded in this study. The most profound limitation is the lack of abundant quality data used to train the models created. The Wolfram dataset used to train the prediction model only consisted of 1,448 cases in a centralized area. The larger GitHub dataset used contained an increased number of datapoints, but with less specific information on each case, limiting the potential prediction capability of models. We were limited by our access to data and relied solely on datasets that were publicly available, yet still based on medical records. Our study and models would improve with direct access to electronic health records, or larger datasets based on them, that contain extensive and detailed accounts of individual COVID-19 cases. Since COVID-19 fatality rates are heterogeneous depending on the region, indicated by the Center for Evidence Based Medicine, additional studies with more representative data would be beneficial. Additionally, the study did not take into account whether patients had received hospital care for COVID-19 treatment prior to their final outcome.

## Conclusion and Future Work

A deep learning model was developed to predict the fatality outcome of an individual who tested positive for COVID-19. In this instance, death is dictated as a rare event and requires a model that accounts for this. Autoencoder proved to be the optimal method to serve as a death prediction model. Additionally, the correlation matrix revealed individuals were at greatest risk of fatality from COVID-19 if they showed respiratory or cardiac based symptoms and were previously diagnosed with a chronic disease.

The model solely predicted death relating to a COVID-19 diagnosis and will be able to provide some guidance to physicians who need to make a decision as to whether or not to admit a person who has tested positive for COVID-19. However, this virus is still capable of having a profound impact on quality of life on infected individuals. In the future, a model should be created that not only predicts death, but also can predict the severity of the progression of the disease. This will prompt individuals to expeditiously seek care, which will prevent the debilitating future dispositions that the disease might induce on the infected individual. This can prevent a large number of people admitted to the ICU if they were to seek care beforehand. Moreover, with increased testing availability in regions, the number of people testing positive for COVID-19 is known for a given region. By incorporating demographic information, health habit (physical excise), or psychology factors, occupation, symptoms and chronic disease of the confirmed case, predictions can be made for the number of required hospitalizations in a given area with the trained model. If this information is combined with the current availability of medical resources deficiencies information (32) in a given area, then proper preparation can be obtained for the amount of medical resources required.

## Data Availability Statement

The datasets presented in this study can be found in an online repository: https://github.com/stccenter/COVID-19/tree/master/prediction/patiant-level%20fatality.

## Author Contributions

YL and HL came up with the original idea. CY advised on the research idea and methods. YL designed the study. YL, MH, JL, and AC conducted the experiments. YL and MH performed the analysis. CY supervised the research and secured the funding source. MH and YL wrote a draft of the manuscript, which was improved by all other authors of the manuscript. All authors contributed to the article and approved the submitted version.

## Conflict of Interest

The authors declare that the research was conducted in the absence of any commercial or financial relationships that could be construed as a potential conflict of interest.

## Footnotes

^1^*“Coronavirus Cases:” Worldometer*. Available online at: www.worldometers.info/coronavirus/?utm_campaign=homeAdvegas1.
